# ARE ENTEROTYPES IN OBESE MODIFIED BY BARIATRIC SURGERY, THE USE OF PROBIOTIC SUPPLEMENTS AND FOOD HABITS?

**DOI:** 10.1590/0102-672020210002e1601

**Published:** 2021-10-18

**Authors:** Giúlia Jager Maximowicz DE-OLIVEIRA, Maria Eliana Madalozzo SCHIEFERDECKER, Antonio Carlos L CAMPOS

**Affiliations:** 1 Nutrition, Universidade Federal do Paraná, PR, Brazil; 2 Departament of Surgery, Universidade Federal do Paraná, Curitiba, PR, Brazil

**Keywords:** Probiotic, Food consumption, Microbiota, Gastric bypass, Obesity, Probiótico, Consumo alimentar, Microbiota, Bypass gástrico, Obesidade

## Abstract

**Introduction:**

Studies suggest that bariatric surgery, use of probiotic supplements and the dietary pattern can change enterotypes, as well as the entire microbial population.

**Objective:**

To verify the influence of bariatric surgery, the use of probiotic supplements and eating habits on enterotypes in obese patients.

**Methods:**

Articles published between the 2015 and 2020 were searched in Lilacs and PubMed with the headings: probiotics, eating behavior, food consumption, food, diet, microbiota, gastrointestinal microbiome, bariatric surgery, gastric bypass and the keyword enterotype in Portuguese, English and Spanish.

**Results:**

Of the 260 articles found, only studies carried out in obese adults relating changes in the enterotype after bariatric surgery or use of probiotics or dietary patterns and original articles were selected. In the end, eight papers on enterotype change and bariatric surgery were selected and categorized, four on the relationship between food consumption and microbiota and one on the effects of probiotics on enterotypes.

**Conclusion:**

The microbial structure is widely modified after bariatric surgery, since the use of probiotic supplement does not bring lasting changes. Enterotypes appear to be shaped by long-term dietary patterns, can modulate how nutrients are metabolized and can be a useful biomarker to improve clinical management.

## RESUMO

**Introdução:**

Estudos sugerem que a cirurgia bariátrica, uso de suplementos probióticos e o padrão alimentar podem mudar enterótipos, assim como toda a população microbiana.

**Objetivo:**

Verificar a influência da cirurgia bariátrica, do uso de suplementos probióticos e de hábitos alimentares nos enterótipos de pacientes obesos.

**Métodos:**

Foi realizada a busca de artigos publicados entre os anos de 2015 e 2020 nas bases de dados Lilacs e PubMed com os descritores: probióticos*,* comportamento alimentar*,* consumo alimentar*,* alimentação*,* dieta*,* microbiota*,* microbioma gastrointestinal*,* cirurgia bariátrica*,* bypass gástrico e a palavra-chave enterótipo em português, inglês e espanhol.

**Resultados:**

Dos 260 artigos encontrados, foram selecionados os estudos originais realizados em adultos obesos relacionando mudanças de enterótipo após cirurgia bariátrica, a padrões alimentares ou ao uso de probiótico. Ao final, foram selecionados e categorizados oito estudos sobre mudança de enterótipo e cirurgia bariátrica, quatro sobre relação entre consumo alimentar e microbiota e somente um sobre efeitos dos probióticos nos enterótipos.

**Conclusão:**

A estrutura microbiana é amplamente modificada após a cirurgia bariátrica. O uso de suplemento probiótico não parece trazer mudanças duradouras. Os enterótipos parecem ser moldados por padrões alimentares em longo prazo e podem modular como os nutrientes são metabolizados, podendo vir a ser um biomarcador útil para melhorar o manejo clínico de pacientes obesos.

## INTRODUCTION

By a multifactorial cause, obesity is closely linked to incorrect eating habits^6^. With the global obesity epidemic, bariatric surgery is now considered the most effective, prompt and long-lasting treatment for patients with morbid obesity^16^. The most performed techniques in the world are Roux-en-Y gastric bypass (RYGB) and vertical gastrectomy^8^.

The intestinal microbiota is colonized by about 100 trillion bacteria which contribute to 3.3 million unique microbial genes and is unique to each individual^21^. After bariatric surgery, eating habits change, patients often use medications and supplements, including probiotics, there is an intense change in body weight, and patients often incorporate healthier lifestyle habits, such as physical activity. All of these factors together have the potential to influence the composition of the intestinal microbiota^21^. 

The evaluation of individual’s microbiota is complex, since it is specific to each one. For this reason, the proposition arose to group intestinal bacteria in groups, called enterotypes^10^. A recent study analyzed faecal metagenomas of individuals from different countries using multidimensional cluster analysis and main components^2^. The authors were able to group faecal metagenomas into three different enterotypes, which were identified by relative amounts of any of the three dominant genera: Bacteroides (enterotype 1), Prevotella (enterotype 2) and Ruminococcus (enterotype 3). Interestingly, these enterotypes appear to be independent of nationality, gender, age or body mass index (BMI).

However, the results of another study^25^ improvement that enterotypes may be strongly associated with the composition of the diet in long term. Enterotype 1, rich in Bacteroides, was strongly associated with the consumption of animal proteins and saturated fats. Enterotype 2, rich in Prevotella, was associated with a carbohydrate-based diet, composed of simple sugars and fibers. Enterotype 3, on the other hand, seems to be strongly related to individual health status^18^. Although it is not known whether enterotypes may be associated with predisposition to certain disease states, these findings have improved that long-term dietary patterns may affect the state of the enterotype, a nutritional-microbiome connection and the pathophysiology in relation to those susceptible to the disease^25^.

Oral administration of supplements with probiotic strains is associated with verified benefits for human health and its use has become usual even after bariatric surgery. Probiotics are living microorganisms that, since they already reside naturally in the healthy human microbiota, when used in an adequate dosage, are safe and confer benefits to the host^19^ ^,^ ^20^.

There is evidence that bariatric surgery induces changes in the intestinal microbiota^1^ ^,^ ^16^ and that each enterotype seems to be strongly associated with food choices, affecting the quantity of specific strains differently in the intestinal microbiota^9^ ^,^ ^19^ ^,^ ^21^. It is also known that the use of probiotics can also modulate the microbiota of the general population^13^. However, studies in the literature that contemplate all of these themes have not been found.

Thus, the aim of this study was to verify the impact of bariatric surgery, the use of probiotics and eating habits on the enterotypes of obese patients.

## METHODS

This is an integrative literature review, accomplished out based on: identification of the theme and selection of the research question, establishment of inclusion and exclusion criteria, identification of pre-selected and selected studies, categorization of selected studies, analysis and interpretation of results and presentation of the review^3^.

The guiding question of this review was: “Can bariatric surgery, the use of probiotic supplements and eating habits affect or modify enterotypes in obese patients?”

The data collection period occurred between the months of May and June 2020 in the Pubmed and Lilacs databases. The following Health Sciences Descriptors (DeCS) and boolean combinations were used: “probiotics”, “eating behavior”, “food consumption”, “food”, “diet”, “microbiota”, “gastrointestinal microbiome”, “bariatric surgery “,” gastric bypass “,” obesity “and the keyword:” enterotype “.

Articles available in portuguese and english, in their entirety, that included changes in enterotypes after bariatric surgery, the use of probiotics or related to dietary patterns were included. Only original articles published and indexed in the referred databases in the last five years were included. Review articles, studies in animals and children and those in which the population was not obese were excluded.

From those selected, data of interest were collected for analysis using a collection instrument that embrace publication, sample, objectives, methodology used and main results of each study. For the tabulation of probiotic studies, in addition to the items previously mentioned, a description of the strains used was added.

Data analysis was performed in a descriptive and comparative way and the studies were categorized into three tables. Those who showed changes in the composition of the intestinal microbiota in individuals undergoing bariatric surgery, studies that related the changes to food consumption and those who studied the use of some probiotic supplementation.

## RESULTS

**FIGURE 1:**
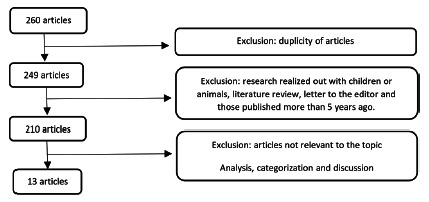
Flowchart of the selection of articles included in the review

After associating the terms, 237 articles were found indexed in the Pubmed database and 23 in Lilacs. After reading the titles, were excluded the duplicates, those that did not meet the inclusion criteria and those that were not relevant to the theme. At the end of the search, 13 articles were selected then analyzed and discussed (Figure 1).

Of the 13 studies, eight were aimed at changing the microbiota after bariatric surgery; four related the enterotypes with eating habits; and one examined the influence of the use of probiotic supplementation on the microbiota.

Studies that evaluated changes in the composition of the intestinal microbiota after bariatric surgery (Table 1) point out that there was a change in enterotypes, an increase in alpha diversity, a relative increase in Proteobacteria, Bacteroidetes and Fusobacteria phyla. Such changes occur from the first three months^4^ ^,^ ^5^ ^,^ ^16^ ^,^ ^17^ post-surgical and can extend for up to 10 years^22^. However, one study^8^ has not found significant changes in the compositions of enterotypes over time.

**TABLE 1:** Changes in the composition of the intestinal microbiota in individuals undergoing bariatric surgery

Authors	Objectives	Population	Main results found
Aron-Wisnewsky, et al., 2019 ^1^	Examine whether the richness of microbial genes get worse in severe obesity and how it relates to worsening comorbidities; and whether the different types of surgery influence microbial characteristics, composition and function.	61 women with severe obesity 41 RYGB and 20 VGB Follow-up before surgery, one, three and 12 months after the procedure	Enterotypes were found in severely obese individuals. A lifestyle and food explain a large part of the microbial composition. There were changes in enterotypes in some patients after surgery (Bacteroidetes 2 to Bacteroidetes 1), such changes occur within 1 year after surgery. The exchange of post-RYGB enterotypes can be an important feature in improving metabolic results
Campisciano et al., 2018 ^5^	Determine whether bariatric surgery shapes the composition of the intestinal microbiota by influencing the intestinal mucosal biofilm and whether this can lead to a positive and long-lasting result	20 obese patients, 10 VG and 10 RYGB 20 eutrophic patients for control Follow-up before and 3 months after surgery	The amount of Proteobacteria decreased after gastric band surgery and increased after RYGB. An increase in the alpha-diversity pattern was observed after 3 months of surgery as well as the predominance of Bacteroidetes, which can improve the immune response to the host and increase the formation of biofilms and the restoration of the microflora balance. It is suggested that characterizing the microbial communities before surgery can help with clinical management.
Campisciano et al., 2018 ^4^	Discussion of data on the microbial composition of patients eligible for bariatric surgery considering the first line of treatment in cases of morbid obesity not responsive to diet and / or physical activity	20 obese patients, 10 VG and 10 RYGB 20 eutrophic patients for control Follow-up before and 3 months after surgery	The microbial composition was dominated by Bacteroidetes, Firmicutes and Proteobacteria despite the fact that Actinobacteria was also detected. In RYGB patients there was an increase in Proteobacteria and Prevotella as well as an improvement in the alpha-diversity pattern after 3 months of surgery, an interesting indirect marker to assess the effectiveness of surgical treatment in terms of restoring the function of the intestinal microflora. It was found that microorganisms adapt quickly to the situation of “hunger” induced by surgery
Dao, et al., 2019 ^8^	Quantify the relative abundance of A. muciniphila before and after 1 year of two types of bariatric surgery in relation to clinical outcomes	65 adult women with severe obesity 21 subjects were followed up before, 1, 3 and 12 months after surgery	The relative abundance of A.muciniphila was lower in severely obese patients when compared to moderate obesity. There was an increase in A. muciniphila after RYGB but not after gastric band. In the enterotype Rumminococcus the A. muciniphila it was significantly increased, in line with its greater richness profile, as well as in the enterotype Bacteroidetes 2, where low levels of A. muciniphila were found. There were no significant changes in the compositions of the enterotypes over time.
Ilhan, et al., 2017 ^14^	Determine microbial differences after RYGB and gastric band surgery; identify the production of metabolites that distinguish the surgeries; reveal relationships between microbiome and weight loss associated with bariatric surgery.	Follow-up of four groups: after RYGB (24), after VGB (14), healthy and eutrophic (10) and morbidly obese (15).	After RYGB surgery there was an increase in Gammaproteobacteria and Fusobacteria. It is suggested that obesity and surgery (RYGB) change the microbial structure and its functions, reflected by the metabolome and that these changes are due to changes in the anatomy of the GIT. The RYGB group had the highest concentration of butyrate and propionate and was not related to the diet.
Palleja, et al., 2016 ^16^	Investigate short and long-term changes in the composition of the microbiota and its functioning after intestinal rearrangement induced by RYGB and changes associated with body weight and metabolism	13 individuals with morbid obesity 5 men and 8 women Before, 3 months and 1 year after RYGB	The change to a healthier metabolism occurs in the first 3 months, when there was an increase in species richness after surgery and was maintained for 1 year. Gene richness tends to increase only after 1 year. Changes in the microbiota, in general, occur within 3 months and remain for up to 1 year. These changes may be related to changes in food preferences
Sánchez-Alcoholado, et al., 2019 ^17^Spain. Methods: We studied 28 patients with severe obesity; 14 underwent a Roux-en-Y gastric bypass (RYGB	Evaluate the short-term evolution of the intestinal microbiome after different bariatric surgery procedures and their functionality and relate it to the resolution of obesity.	28 morbidly obese patients who underwent bariatric surgery using VG or RYGB techniques Data collected before surgery and 3 months after	Just after 3 months of surgery did microbial profiles differ between surgical techniques. After BGYR there was an increase in Proteobacteria and Fusobacteria. It is suggested that pH and bile acid may be the key to the changes produced in the microbiota after bariatric surgery.
Tremaroli, et al., 2015 ^22^	Identify whether changes in the microbiota previously observed in the short term remain stable over time and whether RYGB and gastric band induced specific changes in the intestinal microbiome.	Three groups of women: 7 after 9 years of RYGB, 7 after 9 years of VGB and 7 with severe obesity (control)	There is a significant difference in the composition of the RYGB microbiota versus obese patients. After RYGB, there was an increase in the abundance of Proteobacteria. The bariatric surgery procedure produces a specific change in the microbiota that persists up to a decade after the surgery, being different from the changes related to dietary interventions for weight loss.

RYGB=Roux-en-Y gastric bypass; GIT=gastrointestinal tract; VGB=vertical band gastroplasty; VG=vertical gastrectomy

Regarding the studies that analyzed changes in enterotypes related to food consumption (Table 2), there was a change in enterotypes Prevotella to Bacteroidetes. The consumption of carbohydrates^7,11,19^ and animal protein^12^ ^,^ ^19^ can modify the enterotypes, as well as the type of enterotype can modulate the responses to the diet.

**TABLE 2:** Enterotype changes and their relationship with habits and food consumption

Authors	Objectives	Population	Main results found
Christensen, et al., 2019 ^7^	Specific effects of enterotypes analyzing individuals in a free consumption, with a diet rich in fiber and whole grains or with moderate consumption of fiber and refined wheat. Investigate whether the Prevotella enterotype is associated with other metabolic and intestinal health markers.	70 healthy and overweight adults. 6 weeks duration	Individuals with a high abundance of Prevotella lost weight with a diet rich in fiber and whole grains when compared to a diet based on refined grains and low in fiber. Among these, those with Bacteroidetes enterotype showed less fiber degradation than the dominant Prevotella subjects. This could explain the metabolic difference in response to a high fiber diet.
Hjorth, et al., 2019 ^11^	Reanalysis of a 24-week dietary intervention study for potential differences in the response to weight loss between an individual with low P/B ratio, high P/B and undetectable Prevotella.	80 overweight or obese individuals Deficit of 500kcal in the diet, being the composition of the macronutrients: 30% of lipids, 52% of carbohydrates, 18% of protein	Subjects with a low P/B ratio lost less fat and body weight. The 0-Prevotella group lost more weight. Those with a high P/B ratio were more likely to lose body weight when compared to a low P/B ratio specifically on a diet rich in fiber, carbohydrates, proteins and low fat. It was not possible to conclude whether the P/B ratio is related to different effects of the diet or to an unmeasured marker. The P/B ratio has been proven to be an important biomarker associated with weight loss with diet.
Hjorth, et al., 2020 ^12^	To investigate the interaction between diet, P/B ratio and human salivary amylase gene in weight change, in 26 weeks, as potential pre-treatment markers in personalized nutrition for obesity management	181 participants with increased abdominal circumference One group received a Nordic diet (rich in fiber, whole grains, fruits and vegetables) ad libitum, and the other a controlled diet (Danish diet, similar to the Western diet), for 26 weeks.	The Nordic diet, compared to the Danish diet, had more fiber, whole grains and proteins, less fat and added sugar. Human salivary amylase was not sufficient to predict weight loss between diets. Both participants with a low P/B ratio and those with a high P/B had the same mean of salivary amylase and the enterotypes were not modified. Exclusively among individuals with low salivary amylase and high P/B ratio, they lost more weight with the Nordic diet, while individuals with low P/B ratio lost more weight with the Danish diet.
Shin, et al., 2019 ^19^	Examine the effects of the standard Korean diet and the Western diet on the intestinal microbiota and metabolic profile of healthy Korean adults.	54 obese or overweight Korean participants, before and after 4 weeks of consumption of 3 different diets Typical Korean diet, typical Western (American) diet, recommended healthy American diet Each diet was administered for 4 weeks, with a 2-week intervals for washout.. All participants received the 3 diets.	The type of diet led to changes in microbial communities in relation to the usual diet. After the Korean diet, there was an increase in Firmicutes, a decrease in Bacteroidetes and an increase in the Firmicutes/Bacteroidetes ratio. Among the individuals, 22 belonged to the Bacteroidetes enterotype, 20 to Prevotella and 12 had a balanced relationship between Bacteroidetes and Prevotella. The Korean diet modified the abundance of the phylum Bacteroidetes and increased Firmicutes due to the high intake of carbohydrates and low animal protein. Diet responses were affected by enterotypes, suggesting that it may be a significant variable that contributes to modulating the microbiota.

P/B=Prevotella/Bacteroidetes ratio

In relation to the studies that evaluated change of enterotype with the use of probiotics (Table 3), none were able to observe lasting change. In obese individuals with Prevotella enterotype, the probiotics B. *breve* CBT BR3 and L. *plantarum* CBT LP3 seem to have a beneficial effect^20^.

**TABLE 3:** Enterotype changes in individuals using some probiotic supplementation

Authors	Objectives	Population	Probiotic supplementation	Main results found
Song, et al., 2020 ^20^	To evaluate whether probiotics with multi strains improve markers related to obesity and to investigate different responses by microbial enterotype of the human intestine.	50 healthy obese 12 weeks of intervention, placebo and probiotic group, data collected every 3 weeks	*B. breve* CBT BR3 (15 million viable cells) and *L. plantarum* CBT LP3 (15 million viable cells)	There were no significant differences between groups at the phylum and family level. Anthropometric and biochemical changes were most notable in the Prevotella enterotype. Probiotics may have a beneficial effect on obese individuals with Prevotella enterotype.

## DISCUSSION

According to the findings in the present study, bariatric surgery appears to alter the microbial community. The changes in general occur from the third month on both the RYGB and gastric band techniques^8^ ^,^ ^16^. There is no consensus on the duration of this change. Few studies^16^ ^,^ ^22^ have investigated such changes for more than a year, but one reported that the change produced by the operation is specific to the microbiota and can persist for up to a decade.

In the first three months after bariatric surgery, there was an increase in alpha diversity^5^ and a shift to a healthier metabolism, with an increase in species richness. Genetic richness, on the other hand, seems to increase only one year after the operation^16^. Alpha diversity as it represents the diversity of a habitat or a community of microorganisms and describes the wealth and equality of individuals, can be used as an interesting indirect marker to assess the effectiveness of surgical treatment in terms of restoring the function of intestinal microflora^4^.

After RYGB, there was a reduction in Firmicutes^5^, an increase in Proteobacteria^5^ ^,^ ^16^ ^,^ ^17^ ^,^ ^22^, Prevotella^4^, Bacteroidetes^4^ ^,^ ^5^ ^,^ ^23^, Firmicutes^4^, Fusobacteria^14^ ^,^ ^16^, Actinobacteria^4^, Kleibsiella pneumoniae, Escherichia col^16^, Gammaproteobacteria^14^ and A. muciniphila^8^. The increase in Proteobacteria after RYGB is already well established in the literature and occurs due to changes in environmental, physiological and metabolic conditions^16^. Such changes, as the increase in intestinal oxygen, the growth of facultative anaerobic bacteria^4^ ^,^ ^14^ and the increase in pH facilitate the survival of microorganisms sensitive to acid^14^ ^,^ ^17^, and promote changes in the microbial composition. Thus, microbial changes after RYGB are more due to intestinal rearrangement than to weight loss^16^. Despite this, the abundance of Proteobacteria is not considered beneficial, due to its pro-inflammatory property^22^. 

Regarding the exchange of enterotypes, a study with women after bariatric surgery showed changes from the enterotype Bacteroidetes 2 to Bacteroidetes 1, indicating an important characteristic in the improvement of metabolic results^1^. The Prevotella/Bacteroides (P/B) ratio, it remained stable, with a predominance of Bacteroides^5^.

There was a decrease in Proteobacteria in gastric banding techniques and vertical gastrectomy^5^, besides to maintaining the P/B ratio before and after the operation^4^. In the study accomplished out with 14 patients post vertical gastrectomy a difference was observed in terms of the microbial population with an increase in Verrumicrobia^17^.

Bariatric surgery, especially BGYR, not only decreases the amount of food eaten, but also changes eating behavior and food preferences^15^. In such a way, the changes occurred in the microbiota of these patients may be related to changes in patterns and food preferences^16^ ^,^ ^17^ as well as related to the situation of hunger induced by the operation and the rapid microbial adaptation to such stress^4^.

In the general population, the composition of the intestinal microbiome both at the taxonomic and enterotype levels is directly related to the lifestyle and frequency of ingestion of some foods^1^ ^,^ ^13^ ^,^ ^21^ ^,^ ^25^.

In individuals with a high abundance of Prevotella, greater weight loss was observed with a diet rich in fiber, carbohydrates, proteins and whole grains when compared to a diet based on refined grains and low in fibers^7^ ^,^ ^11^. They also showed greater fiber degradation when compared to subjects with Bacteroidetes enterotype^7^, which can be explained by the fact that high production of short-chain fatty acids affects appetite through different brain signaling pathways, which influence secretion of gastrointestinal hormones.

The P/B ratio was proven to be an important biomarker associated to weight loss with diet^11^. At the same time that the high P/B ratio is associated with a diet rich in resistant starch and fibers^7^, the high-fat and low-fiber diet is associated with a low P/B ratio^11^.

The study that compared the consumption of obese patients for four weeks of a typical Korean diet, a typical American diet, and a recommended American diet, demonstrated that each diet significantly changed the structure of the intestinal microbiota and that responses to diets were affected by the individuals’ initial enterotypes^19^. The traditional Korean diet, composed mainly of high levels of vegetables, whole grains, low levels of animal foods and saturated fat demonstrated a decrease in Bacteroidetes and a significant increase in Firmicutes suggesting that Firmicutes are essential for the dietary metabolism of vegetable polysaccharides^19^.

The observations made show that changes in microbial communities driven by dietary choices may be dependent on the host’s enterotype. It is then suggested that enterotyping provides plausible evidence to be a useful strategy for the development of personalized nutrition^19^.

Despite of the existence of research on the influence of diet on enterotypes probiotics have still been examined for the possibility of impacting enterotypes at a similar level. The use of probiotics to modulate the intestinal microbiota and treat gastrointestinal symptoms has been considered an alternative to improve surgical results^24^.

In Prevotella’s enterotype obese individuals, the probiotics may have a beneficial effect; however, such supplementation has not shown significant differences between groups at the phylum level and microbial family^20^.

Apparently the enterotypes, as well as the entire intestinal microbial population, are shaped mainly by the usual diet, that is, by long-term dietary patterns^21^. Short-term dietary interventions and probiotics appear to cause only transient changes in the microbiota^25^.

There are few studies where food intake has been evaluated along with the use of probiotics and despite being impacting factors no studies have been found that evaluated the association between bariatric surgery, changes in food preferences and the use of probiotics on enterotypes, as well as the literature about the change of enterotypes is limited and not only to other microbial changes.

## CONCLUSION

The intestinal microbiota is modified after bariatric surgery, and this change can influence enterotypes, especially in the first three postoperative months. Both the anatomical as functional modification of the intestinal tract resulting from bariatric surgery and the dietary modifications imposed by the operation seem to be responsible for the change of the enterotypes after it. The use of probiotic supplementation in obese patients was not associated with lasting changes in enterotype. It remains to be investigated whether the use of probiotics after bariatric surgery could influence the enterotype.
